# Leaching, Acute and Chronic Toxicity of Nano-ZnO Incorporated into Photocatalytic Concrete for Pavement Applications Toward *Daphnia magna*

**DOI:** 10.3390/nano16191265

**Published:** 2026-10-07

**Authors:** Fernanda Facin, João Victor Staub de Melo, William Gerson Matias

**Affiliations:** 1 Department of Sanitary and Environmental Engineering, Federal University of Santa Catarina (UFSC), Rua Engenheiro Agronômico Andrei Cristian Ferreira, Trindade, Florianópolis 88040-900, SC, Brazil; fernanda.facin@posgrad.ufsc.br (F.F.); william.g.matias@ufsc.br (W.G.M.); 2 Department of Civil Engineering, Federal University of Santa Catarina (UFSC), Rua Engenheiro Agronômico Andrei Cristian Ferreira, Trindade, Florianópolis 88040-900, SC, Brazil

**Keywords:** nano-ZnO, photocatalytic concrete, pavement, leaching, *D. magna*, aquatic toxicology

## Abstract

Urban air pollution has driven the use of photocatalytic concrete for pavement applications, in which nano-ZnO has emerged as an alternative to nano-TiO_2_. However, its higher solubility raises concerns regarding the release of Zn^2+^ into the aquatic environment. In this context, this study evaluated whether the incorporation of nano-ZnO, at a proportion of 9% relative to the mass of Portland cement, increases the toxicity of pavement concrete leachates toward *D. magna*, compared to conventional concrete. The nano-ZnO powder was physicochemically characterized (XRD, EDXRF, TEM, FTIR, TG/DTG, and colloidal behavior), and the leachates from the conventional and 9% nano-ZnO concretes, obtained by an accelerated TCLP-based leaching of crushed concrete, were characterized by ICP-MS and FTIR. Acute (48 h) and chronic (21 days) toxicity were evaluated for the nano-ZnO powder, for the reference leachate (conventional concrete; data from our previous study), and for the leachate from the nano-modified concrete. ICP-MS indicated that at most ~0.07–0.08% of the total incorporated zinc was leached, and no Zn–O band was detected by FTIR in the freeze-dried leachates, which is consistent with, although it does not prove, the retention of ZnO in the cementitious matrix. In the acute assay, the EC50, 48 h of the nano-modified leachate (10.5% *v*/*v* of the concentrated extract) did not differ significantly from that of the reference leachate (10.4% *v*/*v*; EC50 ratio 1.01, 95% CI 0.87–1.17) and was not attributable to dissolved zinc alone. In a 21-day assay based on OECD TG 211, the nano-modified leachate caused no adverse effect on longevity, size, reproduction, or age at first brood up to the highest concentration tested, whereas the reference leachate caused a slight reduction in size. Overall, nano-ZnO incorporation did not produce additional toxicity toward *D. magna* under the tested conditions.

## 1. Introduction

Urban air pollution, driven mainly by vehicular emissions, represents one of the main public health problems, being associated with thousands of deaths per year worldwide [1]. Despite increasingly stringent emission standards, air quality in large urban centers remains a persistent challenge, which has stimulated the development of pollution mitigation strategies. Among these are photocatalytic concrete pavements, obtained by incorporating photoactive semiconductors into the cementitious matrix, which degrade atmospheric pollutants through advanced oxidation reactions under solar irradiation. In this scenario, nanoscale titanium dioxide is the most widely used semiconductor [1,2,3]. However, zinc oxide (nano-ZnO) has emerged as an alternative, owing to its band gap similar to that of nano-TiO_2_ and its more economical synthesis routes [4,5,6]. Nevertheless, unlike nano-TiO_2_, which is chemically stable and practically insoluble under typical environmental conditions [7], ZnO is an amphoteric oxide whose solubility is sensitive to the pH of the medium, giving this powder a distinct and potentially more complex environmental behavior [8]. Thus, in addition to the possible release of particulate matter through surface wear, the chemistry of the cementitious matrix may govern the speciation and mobility of zinc. In the highly alkaline pore solution of fresh cement paste, the amphoteric ZnO dissolves as hydroxozincate species, which subsequently precipitate as amorphous zinc hydroxide and crystalline calcium hydroxyzincate (CaZn_2_(OH)_6_·2H_2_O) on the surface of the hydrating cement grains, the mechanism responsible for the well-known retarding effect of ZnO on cement hydration [9,10,11]. Once immobilized in these phases and in the C-S-H, zinc can be remobilized as Zn^2+^ when the matrix is exposed to acidic or carbonating conditions, as shown by leaching studies of zinc-bearing cement-based materials [12]. This dissolution-reprecipitation-remobilization pathway constitutes an additional route of toxicological exposure that has so far been examined only indirectly for photocatalytic pavement concretes containing nano-ZnO. Indeed, the available evidence on the release of nanomaterials from photocatalytic cementitious materials refers almost exclusively to nano-TiO_2_, including accelerated leaching and weathering of photocatalytic cement [13], outdoor leaching of titanium from photocatalytic pavements [14], tiered hazard assessment of nano-TiO_2_ photocatalytic cement [15], and the toxicity of leachates from nano-TiO_2_ pavement blocks toward *D. magna* [16]. For nano-ZnO, studies on cementitious composites have focused on hydration, mechanical performance, and photocatalytic efficiency [4,5,6,17], whereas zinc release and its ecotoxicological consequences have been addressed only indirectly, through the leachability of zinc from cement-based materials [12] or the aquatic toxicity of the free nanoparticles [18,19,20].

In this context, recent reviews highlight that weathering processes, surface abrasion (from vehicles and pedestrians), and leaching by rainwater constitute relevant, albeit often underestimated, pathways for the unintentional release of nanomaterials into the aquatic environment [21]. Additionally, environmental risk assessments have already identified nano-ZnO as one of the nanomaterials of greatest relative risk in European freshwaters, when compared to nano-TiO_2_ and nano-Ag, precisely because of its higher solubility and ionic release [22]. Given this scenario, the toxicological evaluation of the fraction of nano-ZnO that is effectively leachable from cementitious matrices intended for pavement applications, rather than of the material in its free form alone, becomes an essential yet still scarce component of toxicological analysis.

The objective of this study was to evaluate whether the incorporation of nano-ZnO, at a proportion of 9% relative to the mass of Portland cement, increases the toxicity of pavement concrete leachates toward *D. magna*, compared to conventional concrete, under accelerated laboratory leaching conditions. For this purpose, the fraction of the nanomaterial mobilized from the hardened cementitious matrix and the acute and chronic toxicity of the leachates to the test organism were characterized. To allow a direct comparison with conventional concrete, the reference-concrete leachate data previously generated by our group under identical conditions and published in [16] were used as the common reference. The mechanical performance and photocatalytic efficiency of pavement blocks nano-modified with ZnO have been reported previously [6,17] and are outside the scope of the present study. In particular, the photocatalytic degradation of NOx by pavement blocks containing nano-ZnO, including the 9% content used here, was demonstrated in [6]; the term “photocatalytic concrete” therefore refers to this previously demonstrated function, which was not re-measured in the present study.

## 2. Materials

### 2.1. Nanometric Zinc Oxide

Nanometric zinc oxide (nano-ZnO) was selected as the photocatalytic semiconductor for this study, given its application in cementitious matrices for heterogeneous photocatalysis [4,5,6]. Material characterization was conducted in two stages: (i) confirmation of crystal structure, size, purity, and morphology, by means of X-ray diffraction (XRD), energy-dispersive X-ray fluorescence spectroscopy (EDXRF), transmission electron microscopy (TEM), Fourier-transform infrared spectroscopy (FTIR), and thermogravimetric analysis (TG/DTG); and (ii) evaluation of the colloidal behavior of the powder in aqueous suspension, relevant to the toxicity assays conducted in this study. Although the powder is a commercial product, it was independently characterized, because manufacturer specifications are nominal and not batch-specific, and because the identity, primary size, and behavior in the test media of the nanomaterial actually tested must be documented in nano-ecotoxicological studies [23]. Table 1 presents the characteristics of the nanometric powder, as reported by the manufacturer (Nanostructured & Amorphous Materials, Inc., Houston, TX, USA) [24].

**Figure 1:**
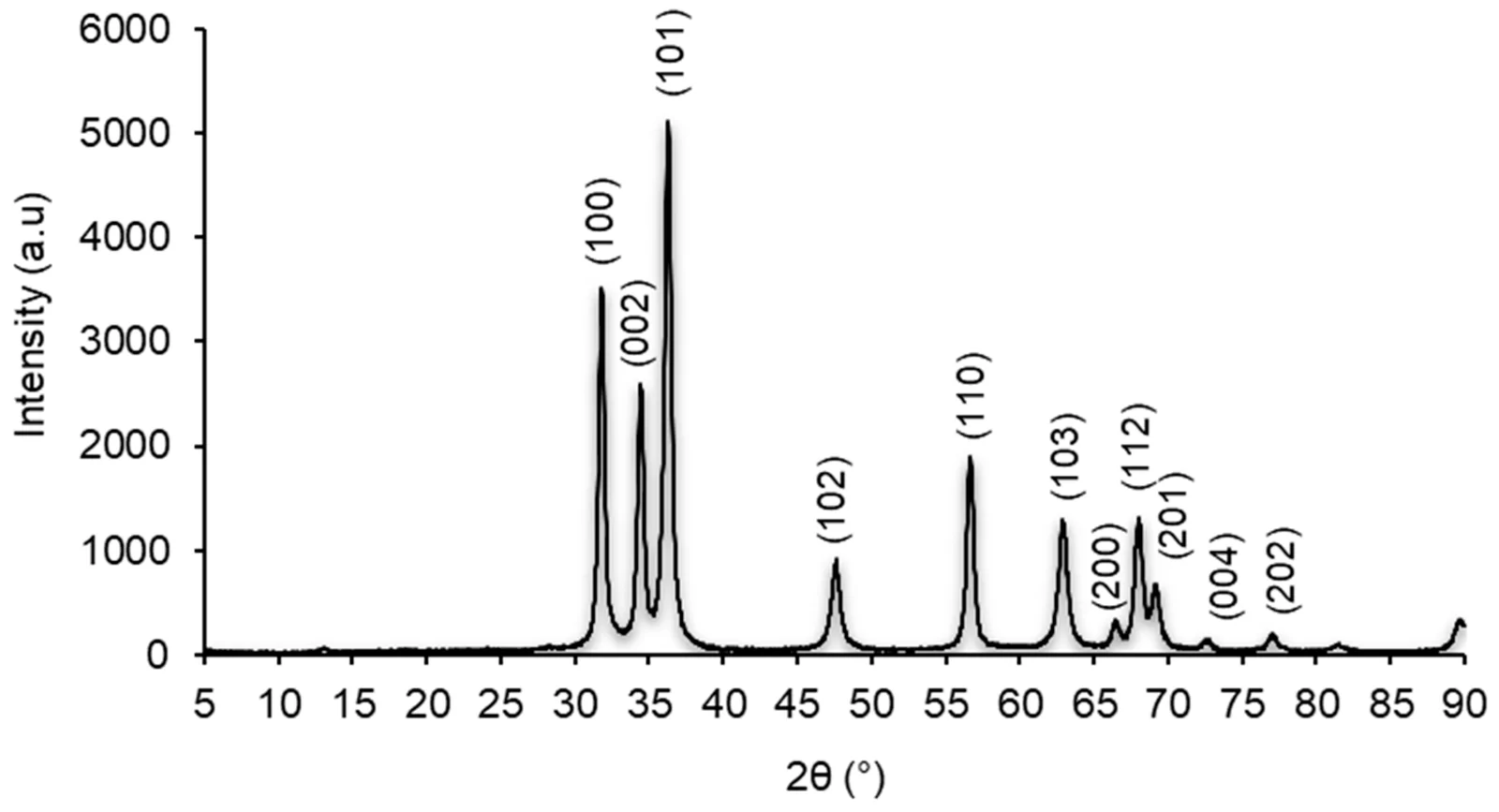
X-ray diffractogram of the ZnO nanoparticles.

First, the crystal structure of ZnO was verified by X-ray diffraction (XRD) on a Rigaku Miniflex II diffractometer, with scans from 5° to 90° (2θ) at a rate of 0.025°/s. The diffractogram (Figure 1) revealed eleven diffraction peaks, three of high intensity [(100), (002), and (101)] and eight additional peaks of lower intensity [(102), (110), (103), (200), (112), (201), (004), and (202)]. All peaks were indexed to the hexagonal wurtzite crystal system (JCPDS 79-2205) [25], with no additional peaks attributable to secondary phases, such as Zn(OH)_2_ or metallic Zn, confirming the formation of single-phase, high-purity ZnO. In addition, the observed intensity sequence (101 > 100 > 002 > 110 > 103 ≈ 112 > 102 > 201 > 200 ≈ 202 > 004) was consistent with the reference pattern, indicating the absence of preferential crystallographic orientation and, therefore, a random distribution of crystallites, typical of polycrystalline samples.

Crystallite size was estimated using the Debye–Scherrer equation [26], D = Kλ/(β·cosθ), with K = 0.9, λ = 1.5406 Å (Cu Kα), and the full width at half maximum (FWHM, β) of each peak obtained by pseudo-Voigt profile fitting [27]. From the two peaks of highest intensity, (101) and (100), values of ~16.5 nm and ~18.8 nm were obtained, respectively, resulting in an average size of ~17.6 nm. When all eleven indexed peaks were considered, the average crystallite size was 15.7 ± 1.7 nm.

The XRD results confirm the formation of ZnO with a hexagonal wurtzite structure, free of detectable crystalline impurities, with lattice parameters very close to bulk values and crystallites within the nanometric range; values consistent with those provided by the manufacturer.

Second, the purity of the nano-ZnO was quantified by energy-dispersive X-ray fluorescence spectroscopy (EDXRF) on a Shimadzu EDX-7000 spectrometer (Shimadzu, Kyoto, Japan), operated with a 10 mm collimator under an air atmosphere, using the standardless quantification method (Quan-FP, Fundamental Parameters). The results (Table 2) confirmed a ZnO content of 98.80 ± 0.14%, with the remainder (1.20%) distributed among five secondary oxides. The presence of these species at trace levels is consistent with residual contaminants typically associated with the industrial production process of the zinc precursor and/or contact with metallic equipment during synthesis and sample handling, and does not indicate the formation of secondary crystalline phases. This interpretation is corroborated by the XRD analysis, which revealed no additional diffraction peaks attributable to these species, since, at such low concentrations (<1%) and possibly in amorphous form or as a solid solution diluted within the ZnO matrix, these elements remain below the detection limit of X-ray diffraction.

**Figure 2:**
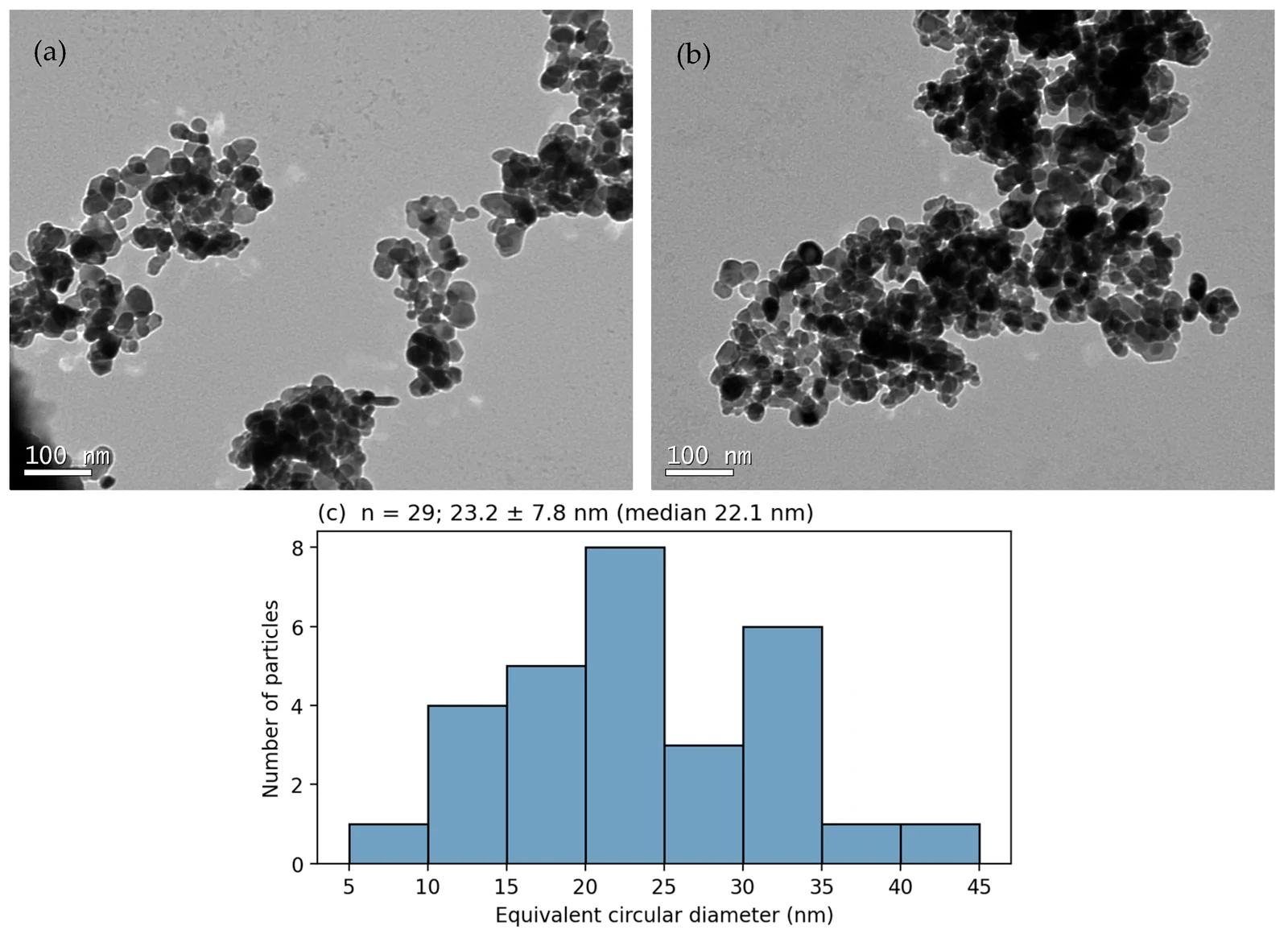
Primary particle size of the nano-ZnO determined by TEM image analysis. (**a**,**b**) Micrographs. (**c**) Distribution of the equivalent circular diameter (23.2 ± 7.8 nm; median 22.1 nm; range 9.7–41.3 nm); the maximum Feret diameter was 28.0 ± 9.4 nm.

Third, particle morphology was investigated by transmission electron microscopy (TEM) on a JEOL JEM-1011 instrument operated at 100 kV. For this analysis, the nanoparticles were suspended in ultrapure water (reverse osmosis, 1 g/L) and sonicated for 2 min at 23% amplitude (115 W, Qsonica Sonicator Q500) to promote deagglomeration of the suspension; an aliquot was then dropped onto carbon-coated copper grids (300 mesh) and dried in a vacuum desiccator for 24 h. The micrographs (Figure 2) confirmed the predominantly spherical to sub-spherical morphology reported by the manufacturer, with particles exhibiting diameters in the nanometric range. The primary particle size was determined by image analysis of the two micrographs (scale calibrated from the 100 nm scale bar, 0.69 nm per pixel): only particles that were fully delimited and did not overlap with neighboring particles were segmented, and their equivalent circular diameter and maximum Feret diameter were computed (scikit-image version 0.26.0, Python 3.12). For the 29 particles that met these criteria, the equivalent circular diameter was 23.2 ± 7.8 nm (median 22.1 nm; range 9.7–41.3 nm) and the maximum Feret diameter was 28.0 ± 9.4 nm, in agreement with the average size of ~20 nm reported by the manufacturer (Table 1) and moderately larger than the crystallite size estimated by XRD (15.7 ± 1.7 nm). The limited number of measurable particles reflects the strong agglomeration of the powder. In addition, despite the ultrasonic treatment applied to promote deagglomeration of the suspension, the micrographs revealed that the nanoparticles remained agglomerated. This behavior is attributed to van der Waals attractive forces, intensified by the high surface-area-to-volume ratio of the nanoparticles.

**Figure 3:**
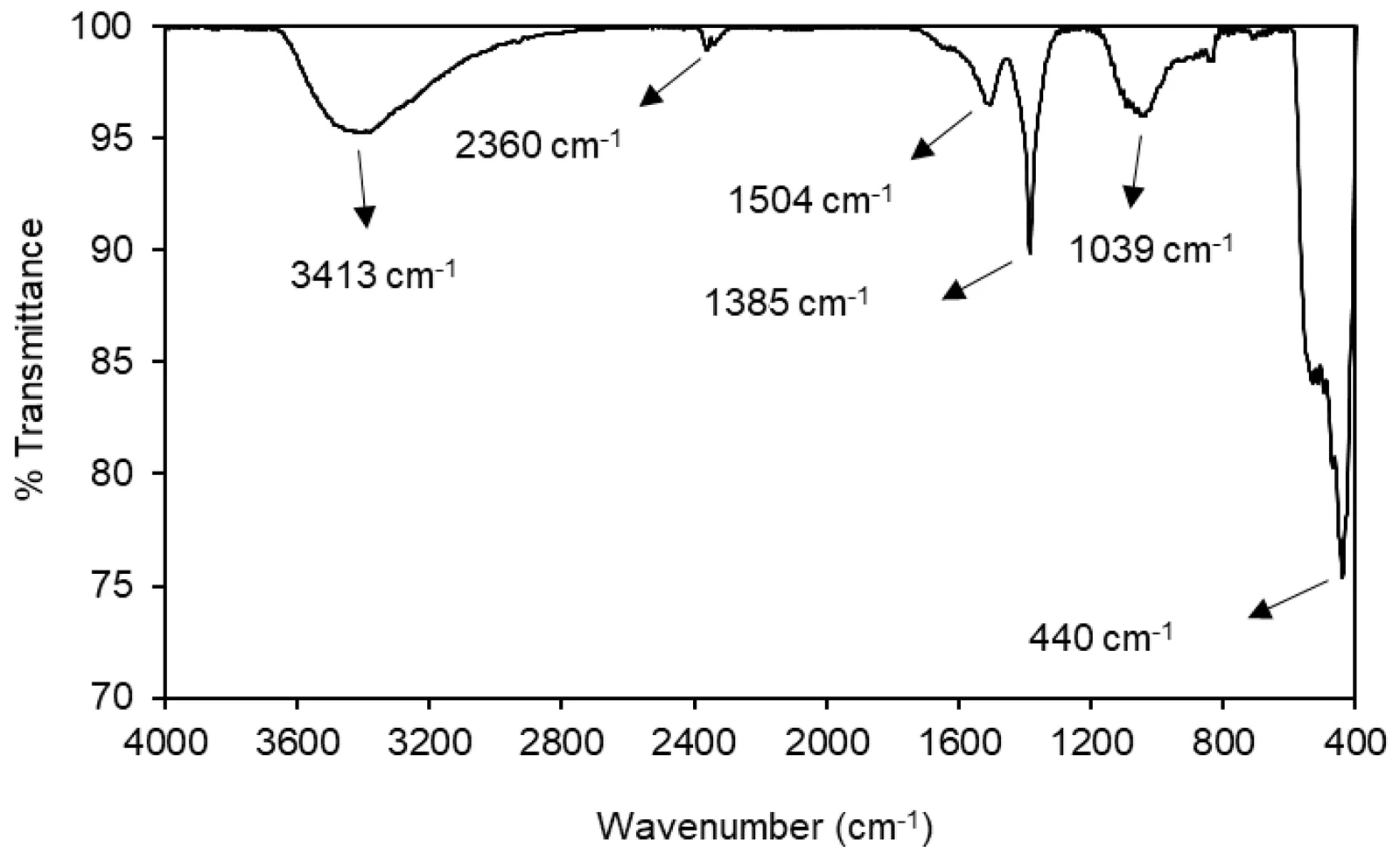
FTIR spectrum of the ZnO nanoparticles.

Fourth, Fourier-transform infrared spectroscopy (FTIR), performed on an Agilent Technologies Cary 660 spectrometer, was used to confirm the chemical nature of the nano-ZnO and to identify adsorbed surface species. The spectrum (Figure 3) showed an intense, broad band in the 400–530 cm^−1^ region, with a maximum at 440 cm^−1^ and shoulders at ~469, 498, 513, and 528 cm^−1^, attributed to Zn–O stretching in the wurtzite lattice; its multicomponent structure reflects the different lattice vibrational modes allowed by the hexagonal symmetry of the crystal [28]. A second broad band of moderate intensity, centered at 3413 cm^−1^, was attributed to O–H stretching of hydroxyl groups and water molecules adsorbed on the nanoparticle surface [29]. The sharp band at 1385 cm^−1^ and the weaker band at 1504 cm^−1^ were assigned to the two components of the asymmetric C–O stretching mode (ν_3_) of carbonate species chemisorbed on the oxide surface, whose degeneracy is lifted upon coordination to surface Zn^2+^ sites; these species result from the uptake of atmospheric CO_2_ during synthesis, processing, or storage [30]. The weak, broad band at ~1039 cm^−1^ is consistent with the symmetric C–O stretching mode (ν_1_) of the same coordinated carbonate, which is infrared-inactive in the free ion but becomes weakly active when its symmetry is lowered by surface coordination. A weak band at 2360 cm^−1^ was attributed to the asymmetric stretching of gaseous CO_2_ in the optical path of the instrument and is unrelated to the composition of the sample [31]. Overall, the FTIR spectrum is consistent with wurtzite ZnO, shows no bands attributable to residual organic groups, and indicates that the only relevant adsorbed surface species are water and small amounts of carbonate. Band positions are reported as integer wavenumbers, consistent with the spectral resolution of the measurement.

**Figure 4:**
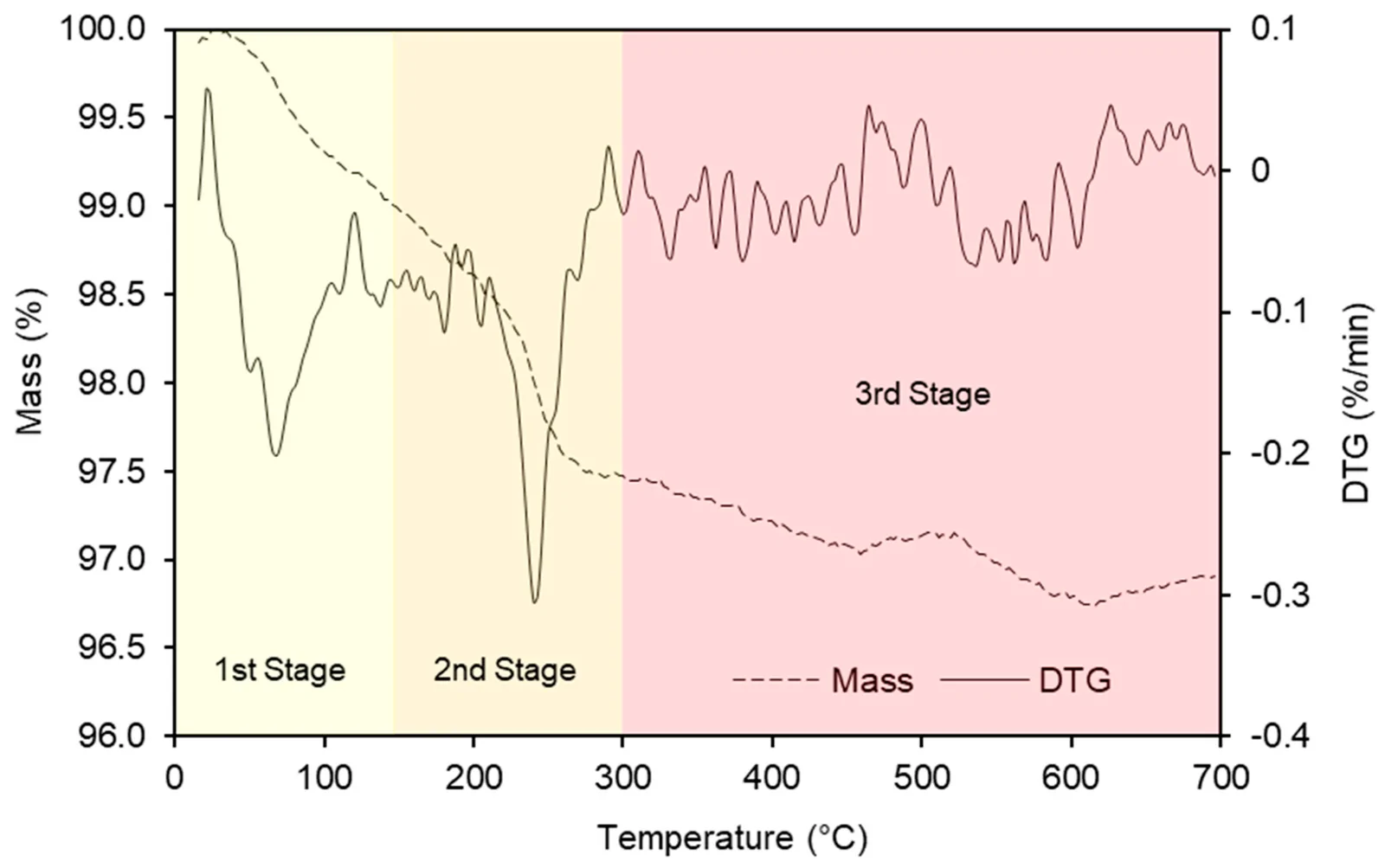
Thermogravimetric analysis of the ZnO nanoparticles.

Finally, the thermal stability of the nano-ZnO was evaluated by thermogravimetric analysis (TG/DTG) on an STA 449 F3 Jupiter–Netzsch analyzer, using approximately 10 mg of sample heated to ~700 °C at a rate of 10 °C/min, as shown in Figure 4. The thermogravimetric curve revealed a three-stage decomposition profile, with a total mass loss of ~2.98%. In the first stage, between room temperature and ~150 °C, a mass loss of ~0.93% was observed, attributed to the elimination of physically adsorbed water on the nanoparticle surface [32]; an event consistent with the O–H stretching band identified by FTIR at 3413 cm^−1^. In the second stage, between 150 °C and 300 °C, the loss rate intensifies, with the DTG curve peak centered at 240.2 °C (−0.305%/min), accounting for ~1.51% of the total mass loss. This event is attributed to the combined action of dehydroxylation of more strongly bound surface hydroxyl groups (chemically adsorbed water) and the thermal decomposition of surface carbonate species (CO_3_^2−^) [33], whose presence was identified by FTIR through the ν_3_ bands at 1385 and 1504 cm^−1^. Above ~300 °C, the loss rate decreases substantially (<0.07%/min; ~0.54%) until the end of the test, possibly associated with the decomposition of more thermally stable carbonate/hydroxide species or the dehydroxylation of –OH groups located structurally deeper within the crystalline surface [34]. The total mass loss observed (~2.98% up to 700 °C) is consistent with the manufacturer’s specification (≤3% at 850 °C/2 h).

These results are consistent with those reported for other wurtzite ZnO nanopowders. Motelica et al. [35], for instance, also obtained single-phase wurtzite by XRD, assigned the Zn–O stretching to absorptions below 500 cm^−1^ (457 and 410 cm^−1^, compared with 440 cm^−1^ here) and the broad band near 3400 cm^−1^ (3413 cm^−1^ here) to surface hydroxyl groups, and observed weak bands near 1033 cm^−1^, a region in which the present powder also shows a weak band (1039 cm^−1^). In that study, the TEM particle sizes (34–54 nm) agreed with the crystallite sizes, indicating monocrystalline particles, whereas here the TEM diameter (~23 nm) was moderately larger than the XRD crystallite size (~16 nm), suggesting that some particles comprise more than one coherently diffracting domain or that peak broadening includes a microstrain contribution. The main difference concerns the surface species: the solvothermally synthesized powders of Motelica et al. [35] retained acetate (carboxylate bands near 1435 and 1568 cm^−1^, decomposing at ~260–290 °C), whereas the commercial powder used here showed only carbonate bands (1385 and 1504 cm^−1^) and no carboxylate or other organic residues, in line with its TG/DTG profile, in which the main event (DTG peak at 240 °C) was attributed to dehydroxylation and carbonate decomposition, and with its total mass loss (~2.98%), which is within the manufacturer’s specification.

In summary, the combined results (XRD, EDXRF, TEM, FTIR, and TG/DTG) validate the size, structure, purity, and crystallographic quality of the nanometric material.

Since solid-state characteristics do not necessarily predict behavior in a liquid medium, the colloidal suspension of the nanoparticles was characterized in terms of zeta potential, effective diameter, polydispersity index, and pH, both in ultrapure water and in the test media used with *D. magna* (ISO for acute toxicity; M4 for chronic toxicity; compositions in Section 3.3). The suspensions were prepared at 100 mg/L, dispersed by ultrasonication (Qsonica Q500, 115 W, 2 min), with pH measured at 20 ± 1 °C (Alfakit AT-355) and zeta potential, effective diameter, and polydispersity index measured in triplicate by light scattering (Nanobrook 90Plus PALS, Brookhaven). The results are presented in Table 3.

According to Table 3, all three media exhibited a zeta potential magnitude below ±30 mV (a reference commonly used as an indicative threshold for the electrostatic stability of colloidal suspensions [36]); that is, in none of the three media could the nano-ZnO suspension be considered electrostatically stable against aggregation. This trend is more pronounced in ISO (−2.20 mV) and M4 (−1.25 mV), media in which the zeta potential approaches zero, a condition associated with lower electrostatic repulsion between particles and, therefore, a greater propensity for aggregation. In ultrapure water, although the value (11.20 mV) is still far from the stability threshold, the suspension exhibits a more defined surface charge than in the test media; behavior consistent with compression of the electrical double layer around the particles by the dissolved salts in the ISO and M4 media, an ionic-strength effect described by the Derjaguin-Landau-Verwey-Overbeek theory for ZnO nanoparticles in aqueous media [8]. This picture is corroborated by the effective diameter values (517.65 nm in ultrapure water, 1690.11 nm in ISO, and 1121.55 nm in M4), which substantially exceed both the crystallite size determined by XRD (~15.7 nm) and the primary particle size measured by TEM (23.2 ± 7.8 nm). This increase relative to the primary size, associated with the near-neutralization of the zeta potential, indicates that the salts present in the test media favor aggregation of the nanoparticles in suspension, consistent with what had already been qualitatively observed by TEM in the solid state (Figure 2). The polydispersity indices (0.12 [ultrapure water]; 0.26 [M4]; and 0.27 [ISO]) indicate moderately heterogeneous suspensions in terms of size distribution (slightly more dispersed in ISO and M4 than in ultrapure water).

From a toxicological standpoint, the effective diameters observed in the media at 100 mg/L (517.65 to 1690.11 nm) fall within the range of particles capturable by the filtering apparatus of *D. magna* [37], so that the observed aggregation pattern should not, by itself, restrict ingestion of the nano-ZnO by the test organism.

### 2.2. Hydraulic Binder and Mineral Aggregates

The hydraulic binder and mineral aggregate used in this study are the same as those employed in previous research by the group [16]. The binder consists of a pozzolan-blended Portland cement, composed of clinker and gypsum (76 to 94%), limestone (0 to 10%), and pozzolan (6 to 14%), while the mineral aggregate is of granitic origin.

## 3. Methods

**Figure 5:**
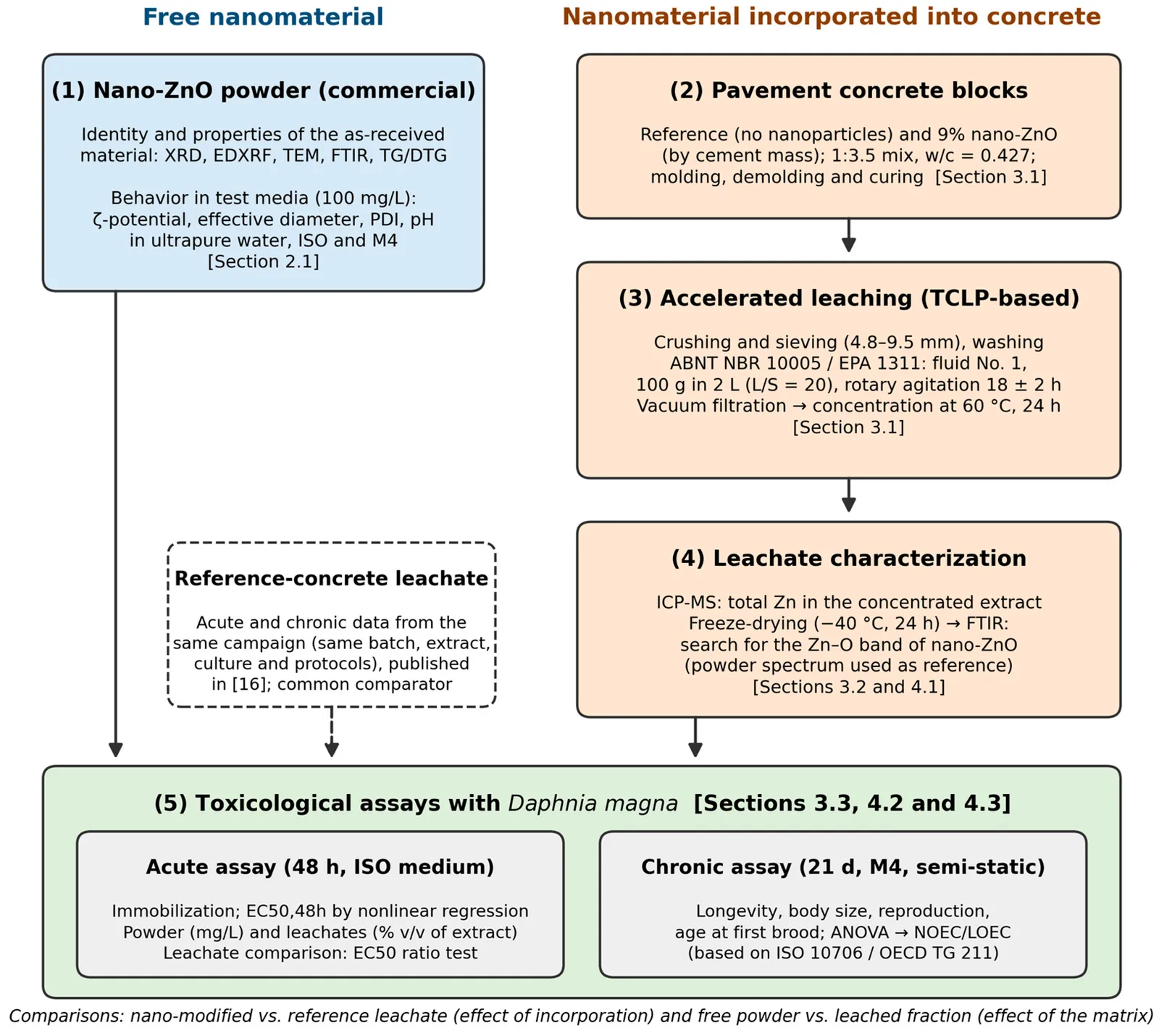
Flowchart of the experimental program, indicating the sequence of the experiments, the sections in which each stage is described, and the comparisons supported by the study design.

The experimental program comprised the following stages: molding of the concrete test specimens and leaching, characterization of the leachate extracts, and toxicological assays with *D. magna*. The sequence of the experiments and the comparisons that they support are summarized in Figure 5.

### 3.1. Molding of Concrete Test Specimens and Leaching

Concrete blocks for pavement applications were molded and divided into two groups: a reference group, without the addition of nanoparticles, and a nano-modified group, with nano-ZnO incorporated at 9% relative to the mass of Portland cement. The concrete was produced with a 1:3.5 mix ratio (cement:mineral aggregate) and a water/cement ratio of 0.427. The specimens were molded in metal molds with the aid of a hydraulic press, demolded, and cured in accordance with ASTM C192/C192M [38]. The mechanical performance (compressive strength, flexural strength, and dynamic modulus of elasticity) and the microstructure of pavement blocks nano-modified with 3, 6, and 9% ZnO were characterized in a previous study by our group [17].

**Figure 6:**
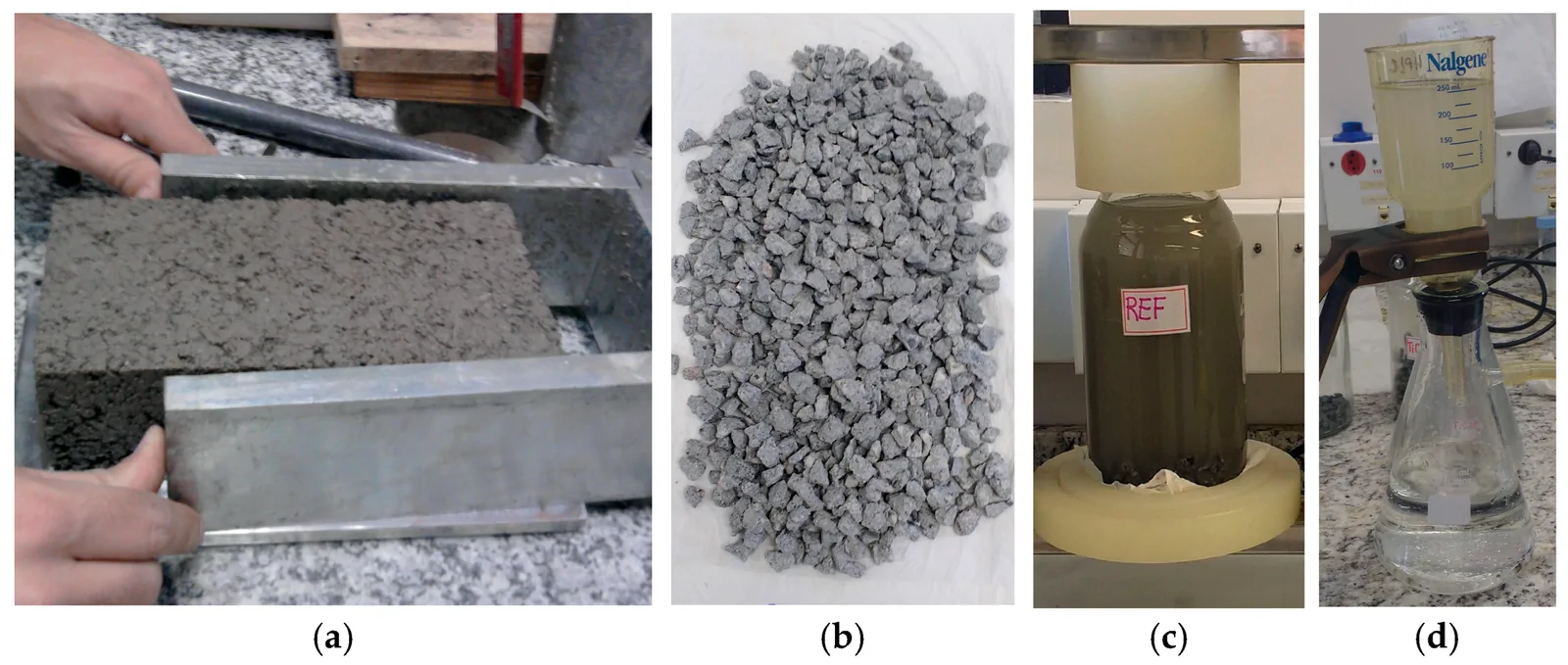
(**a**) Molding of a pavement concrete block, (**b**) concrete fragments (4.8 mm to 9.5 mm), (**c**) sample in a rotary bottle agitator, (**d**) filtration of the extract.

The leaching process was conducted in accordance with ABNT NBR 10005 [39], based on the Toxicity Characteristic Leaching Procedure (TCLP, EPA SW-846, Method 1311) [40], on which the Brazilian standard is based. The concrete samples (pavement concrete blocks) were crushed and sieved to a particle size range of 4.8 mm to 9.5 mm, and washed in ultrapure water to remove fine particulate material generated during crushing. Extraction fluid No. 1 was used, prepared with 5.7 mL of glacial acetic acid, 64.3 mL of 1.0 N sodium hydroxide, and deionized water to a final volume of 1 L. For each assay, 100 g of solid sample, corresponding to the 4.8–9.5 mm particle size fraction, was immersed in 2 L of the extraction fluid in bottles sealed with PTFE tape. The bottles were subjected to rotary agitation at 30 ± 2 rpm for 18 ± 2 h, at a temperature of up to 25 °C. After this period, the extract was filtered using a vacuum apparatus, followed by concentration in an oven at 60 °C for 24 h, which reduced the extract volume from 2 L to approximately 1.6–1.8 L, corresponding to a concentration factor (CF) of approximately 1.11–1.25. All subsequent analyses (ICP-MS and FTIR) and all toxicity tests were performed on this same concentrated extract; therefore, the % *v*/*v* dilutions and the Zn concentrations reported hereafter refer to it. Where relevant, the results are also expressed as original-leachate equivalents, by dividing concentrations by the CF (Zn) or multiplying % *v*/*v* values by the CF (EC50). The samples were kept under refrigeration until the analyses were performed. Figure 6 illustrates this stage of the study.

It should be emphasized that TCLP was developed to estimate the mobility of constituents under a co-disposal (landfill) leaching scenario [40], and not to reproduce the wash-off of in-service pavement surfaces by rainfall. In the present study, it was therefore adopted as an accelerated, standardized, and conservative leaching condition, for three reasons: (i) crushing the blocks into 4.8–9.5 mm fragments (mandatory in TCLP for monolithic samples) greatly increases the specific surface area in contact with the fluid and exposes internal, cement paste, whereas in service only the surface layer is exposed; (ii) the acetate-buffered extraction fluid is more aggressive than typical rainwater, and its neutralization by portlandite and C-S-H promotes the dissolution of matrix phases; and (iii) the high liquid-to-solid ratio and continuous agitation maximize mass transfer. Consequently, the leachates tested here should be regarded as an upper-bound laboratory representation of the dissolved/colloidal fraction mobilizable from the matrix, and not as a direct surrogate of pavement runoff. Surface-controlled release from monolithic pavements would be more appropriately assessed by tank or dynamic surface leaching tests [41,42] or by field runoff monitoring [14].

### 3.2. Leachate Characterization

The leachate extracts were characterized using two complementary analytical techniques, employed to evaluate, respectively, the fraction of zinc released and the possible release of particulate material from the cementitious matrix. The first technique, inductively coupled plasma mass spectrometry (ICP-MS; Elan 6000 model, PerkinElmer SCIEX, Concord, ON, Canada), with sample introduction via pneumatic nebulizer, was used to quantify the zinc (Zn) concentration in the concentrated leachate extracts (n = 3 determinations). The samples were previously acidified with nitric acid (HNO_3_) to pH < 2, following the standard procedure for the preservation and stabilization of metals in aqueous samples [43]. It is important to note that ICP-MS does not distinguish between dissolved Zn^2+^ and any ZnO nanoparticles or small aggregates able to pass through the filter and remaining in suspension in the extract; thus, the quantified zinc concentration represents the total elemental content in the sample, and not exclusively the free ionic fraction.

**Figure 7:**
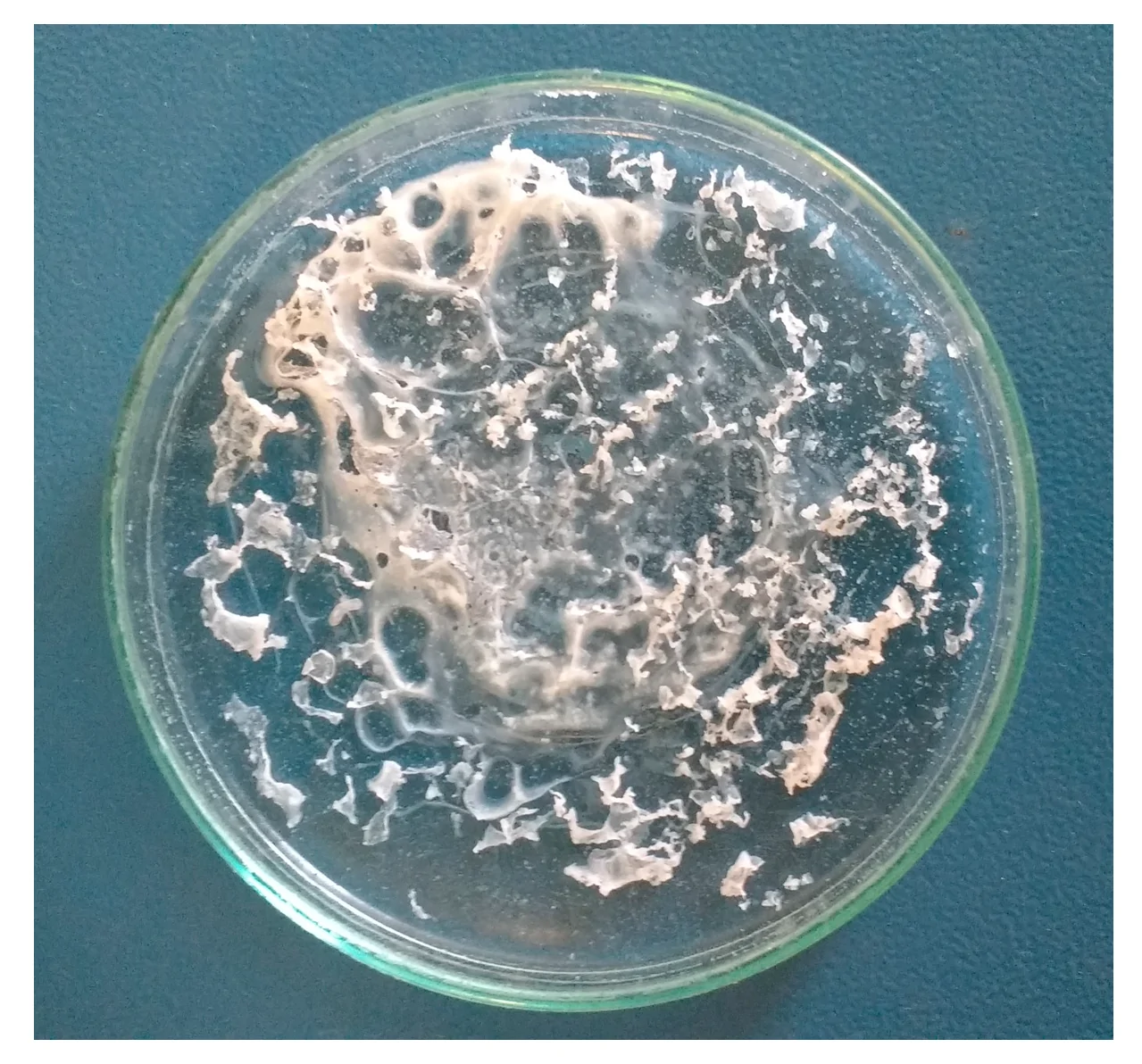
Solid sample of the vacuum freeze-dried leachate.

The second technique, Fourier-transform infrared spectroscopy (FTIR; Agilent Cary 660 spectrometer, Agilent Technologies, Santa Clara, CA, USA), aimed to verify any possible detachment of ZnO nanoparticles from the cementitious matrix and their migration into the liquid extract, through identification of the characteristic Zn–O stretching band in the leachate spectrum. Considering that any traces of ZnO in the leachate extract might be present at low concentrations, the extracts were freeze-dried under vacuum (−40 °C for 24 h in an L101—Liotop freeze-dryer, Liotop, São Carlos, SP, Brazil) prior to FTIR analysis (Figure 7), in order to concentrate the solid residual material and increase detection sensitivity. For comparison purposes, the spectrum of the nano-ZnO powder (Figure 3) was used as a spectral reference for identifying coincident bands in the freeze-dried extracts. Because the detection capability of FTIR for a minor crystalline component dispersed in a complex, salt-dominated solid residue is limited, the absence of a band can only indicate that the corresponding phase, if present, was below this detection capability.

### 3.3. Toxicological Assays with D. magna

Neonates of *D. magna* (2–26 h old) were obtained from females aged 10–60 days, kept in continuous culture according to ABNT NBR 12713 [44] and ISO 6341 [45]. Cultures were maintained in M4 medium at 20 ± 2 °C under a 16 h light:8 h dark photoperiod and fed the green alga *Desmodesmus subspicatus* (formerly *Scenedesmus subspicatus*), supplied at each renewal of the culture medium, which was performed three times per week, when the number of adult organisms was checked and the vessels were cleaned. Dissolved oxygen in the cultures remained above 3 mg/L and pH remained within the range of 6–9. Culture sensitivity was checked weekly using potassium dichromate (K_2_Cr_2_O_7_) as the reference toxicant, and only cultures whose 24 h EC50 fell within the acceptance range of 0.6–1.7 mg/L established by ABNT NBR 12713 [44] were used in the tests.

Two reconstituted media were used, each according to the respective test guideline. The acute assays were performed in ISO medium, the dilution water prescribed by ISO 6341 [45], which contains only CaCl_2_·2H_2_O, MgSO_4_·7H_2_O, NaHCO_3_, and KCl, with no trace metals or chelating agents. The cultures and the chronic assay were performed in Elendt M4 medium, prepared as specified in OECD TG 211, Annex 2 [46], which contains the same major salts supplemented with silicate, nitrate, phosphates, trace elements, vitamins, and EDTA, required to sustain growth and reproduction over 21 days.

Three test substances were evaluated: (i) the leachate from the reference concrete (without nanoparticles); (ii) the leachate from the nano-modified concrete containing 9% nano-ZnO; and (iii) the suspension of pure nano-ZnO powder. The inclusion of this last test substance allows the effect of the material in its free form to be distinguished from the effect of the material once incorporated and subsequently leached from the cementitious matrix; a comparison that is central to evaluating whether the incorporation process in concrete attenuates, maintains, or enhances the intrinsic toxicity of the nanomaterial.

The acute and chronic data for the reference-concrete leachate were generated in the experimental campaign described in our previous study on nano-TiO_2_ [16], using the same concrete batch, leaching procedure, extract, *D. magna* culture, and test protocols, and have already been published there. They are reproduced here, with citation, only as the common reference for comparison and are not presented as new results.

In the acute assay, neonates were exposed for 48 ± 1 h to the test substances diluted in ISO medium, in accordance with ABNT NBR 12713 [44], in duplicate, with 10 organisms per container (20 per dilution, which meets the minimum of 20 animals per concentration required by ISO 6341 [45] and OECD TG 202 [47]), to determine the effective concentration capable of immobilizing and/or killing half of the exposed population (EC50, 48 h). The two leachates were tested at dilutions of 4.2%, 6.25%, 10%, 20%, 50%, and 100%, in addition to the control with pure ISO medium. The ZnO nanoparticles, in turn, were tested at concentrations of 1, 5, 10, 15, 20, and 25 mg/L, in addition to the control (a range defined after preliminary screening tests, which indicated high toxicity of the material already at low concentrations, making it unnecessary to test the range of hundreds of mg/L). All nanoparticle suspensions in ISO medium were homogenized by ultrasonication (115 W, 2 min). At the end of the exposure period, immobile and/or dead organisms were counted at each dilution, and the EC50, 48 h of each test substance was estimated by nonlinear regression (log(agonist) vs. normalized response; variable slope) in GraphPad Prism^®^ software (Version 11.1.0). Because overlapping confidence intervals do not constitute a formal statistical test [48], the EC50, 48 h values of the two leachates were compared by the ratio test of Wheeler et al. [48]: the 95% confidence interval (CI) of the EC50 ratio was calculated from the standard errors of the log-transformed EC50 estimates, and the two values were considered significantly different when this interval did not include 1. As a complementary indication of similarity, it was examined whether the 90% CI of the ratio fell entirely within a conventional margin of 0.80–1.25 (two one-sided tests approach) [49]. For descriptive purposes, and to ensure comparability with our previous study [16] and with the nano-ecotoxicology literature, in which these schemes are still widely used, the EC50, 48 h of the nano-ZnO powder, expressed as mass concentration (mg/L), was categorized according to the criteria of the European Commission Directive 93/67/EEC [50]: EC50, 48 h below 1 mg/L indicates a very toxic material; between 1 and 10 mg/L, toxic; between 10 and 100 mg/L, harmful; and above 100 mg/L, not classified for aquatic organisms. The EC50, 48 h values of the leachates, after conversion into original-leachate equivalents (% *v*/*v* of the concentrated extract × CF; Section 3.1), in turn, were classified according to the analogous scheme proposed by Tonkes et al. [51], specific to effluents/leachates: EC50, 48 h below 1% indicates very high toxicity; between 1 and 10%, moderate toxicity; between 10 and 100%, slight toxicity; and above 100%, non-toxic. Because these schemes are not current regulatory frameworks, the result for the powder was also placed within the acute aquatic hazard categories of the UN Globally Harmonized System (GHS) [52] and of the EU CLP Regulation [53], and the results for the leachates were additionally expressed as acute toxic units (TUa = 100/EC50, 48 h, with EC50, 48 h in % *v*/*v* of the original leachate; values referred to the concentrated extract are also reported for transparency). These categorizations describe intrinsic toxicity under laboratory conditions only; they do not constitute a regulatory classification or an environmental risk assessment, which would also require a realistic characterization of exposure.

**Figure 8:**
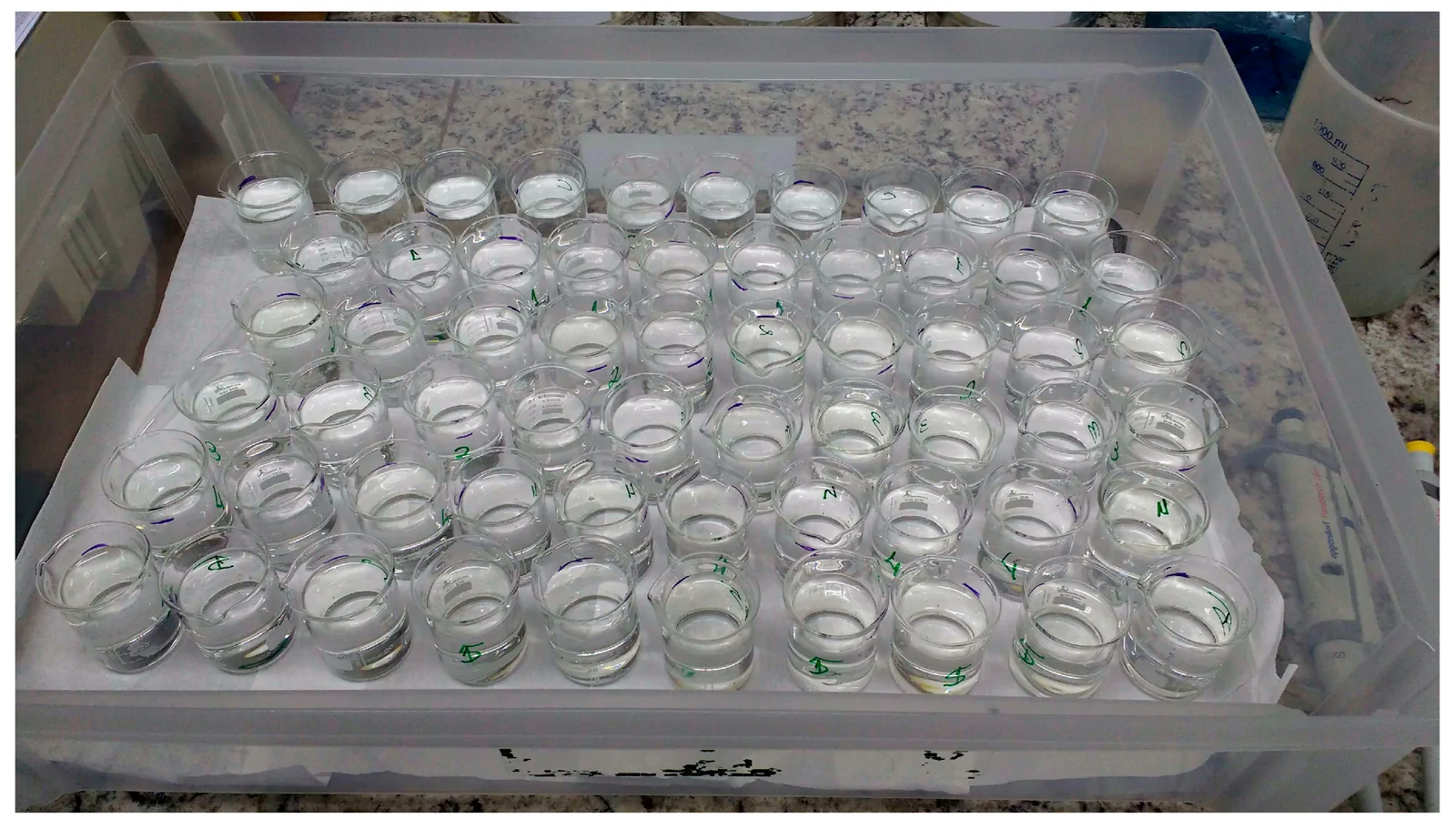
Setup of the chronic toxicity assay with *D. magna*, with organisms individually housed in 50 mL beakers.

The chronic assay was based on ISO 10706 [54] and OECD Test Guideline No. 211 [46] (deviations from the guideline are listed below), with neonates exposed for 21 days in individual replicates (1 organism per 50 mL glass beaker, 10 replicates per concentration), in M4 medium (Figure 8), under semi-static conditions. The test solutions were renewed three times per week; the test was kept in the same temperature-controlled incubator as the cultures, at 20 ± 2 °C, under diffuse illumination with a 16 h light:8 h dark photoperiod. Four parameters were evaluated at the end of the period: longevity, body size (length), reproduction (expressed as neonates per brood), and age at first brood. Table 4 presents the concentrations used in the chronic toxicity assays.

The chronic assay deviated from OECD TG 211 [46] in one main respect, which is considered in the interpretation of the results (Section 4.3) and in the limitations of the study (Section 4.4): reproduction was recorded and analyzed as neonates per brood, rather than as the total number of living offspring per surviving parent at day 21, on which the guideline’s reproduction validity criterion (≥60 offspring) is based.

The results of the chronic assay were subjected to one-way analysis of variance (ANOVA, GraphPad Prism^®^, significance level *p* < 0.05), comparing each concentration to the control, preceded by verification of normality and homogeneity of variances. The lowest concentration with a statistically significant difference relative to the control was defined as the lowest observed effect concentration (LOEC); the concentration immediately below this, as the no observed effect concentration (NOEC). In line with OECD TG 211 [46], (i) statistically significant increases in reproduction were not treated as adverse effects for NOEC/LOEC derivation, and (ii) an isolated significant difference not accompanied by an equal or greater effect at higher concentrations was not assigned as the LOEC; such cases are reported and discussed separately.

## 4. Results and Discussion

### 4.1. Leachate Characterization

**Figure 9:**
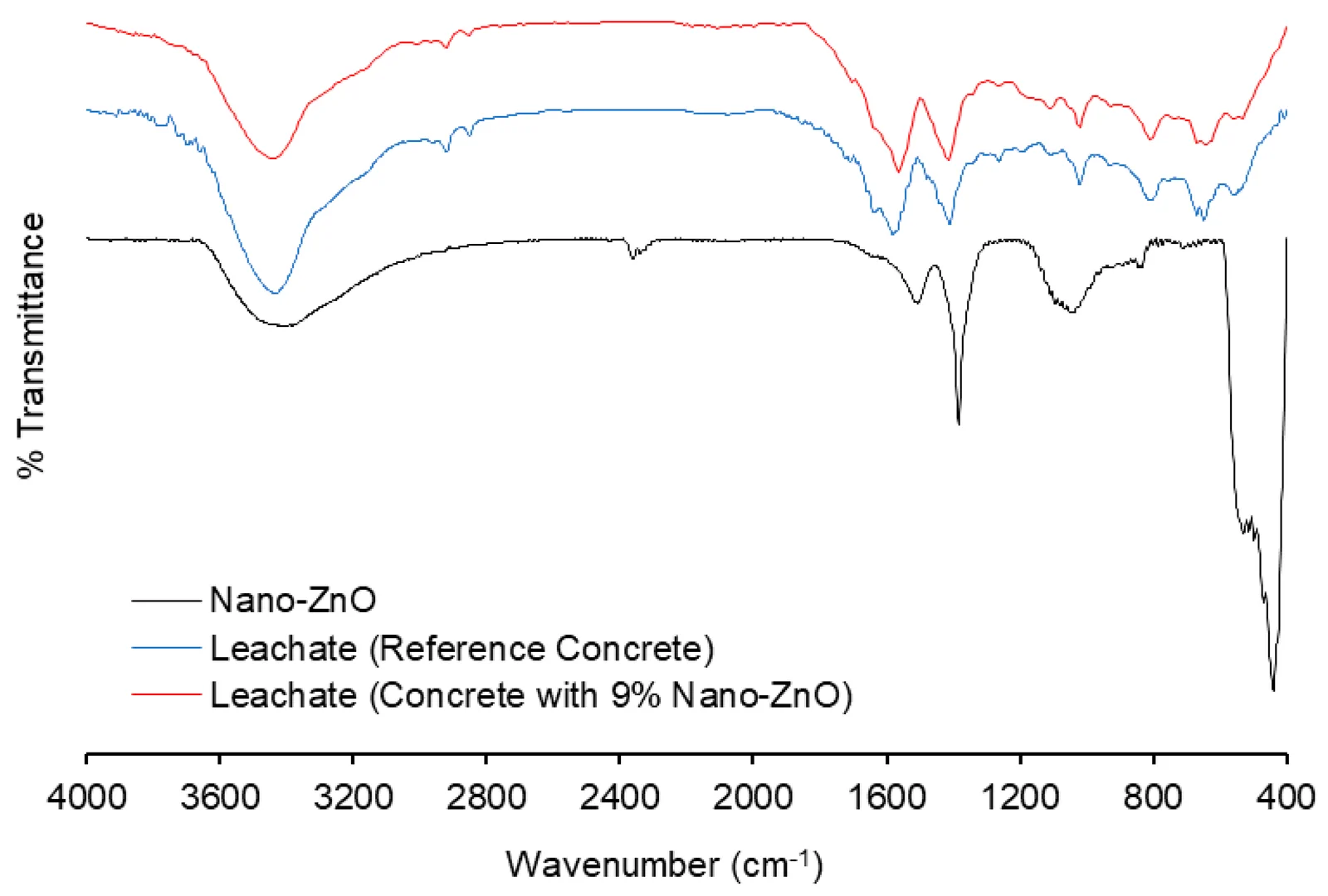
FTIR spectra of the leachate extracts (freeze-dried) and of the pure nano-ZnO powder.

Zinc quantification by ICP-MS revealed a total Zn concentration of 653 ± 76 µg/L in the concentrated leachate from the nano-modified concrete containing 9% nano-ZnO, compared with a non-detected result in the leachate from the reference concrete; an expected result, since the latter does not contain zinc in its formulation. To contextualize the magnitude of this result relative to the total amount of nano-ZnO incorporated into the concrete, a mass-balance estimate was performed. Considering the 1:3.5 mix ratio (cement:aggregate) and the addition of nano-ZnO equivalent to 9% of the cement mass, it is estimated that nano-ZnO corresponds to approximately 1.8–2% of the total mass of the hardened concrete. For a 100 g sample of crushed concrete (the mass effectively used in the leaching assay), this corresponds to approximately 1.8–2 g of ZnO, or 1.4–1.6 g of elemental zinc (considering the mass fraction of Zn in ZnO, MM = 81.38 g/mol). Because the ICP-MS determination was carried out on the extract after its concentration at 60 °C, the leached Zn mass was calculated from the measured concentration (653 µg/L) and the final volume of the concentrated extract (~1.6–1.8 L; Section 3.1), giving ~1.0–1.2 mg of zinc, which corresponds to a leached fraction of approximately 0.07–0.08% of the total zinc present in the material; the equivalent concentration in the original TCLP leachate (2 L; liquid-to-solid ratio of 20:1) was ~520–590 µg/L. Even an upper-bound calculation that ignores the concentration step (653 µg/L × 2 L ≈ 1.3 mg) yields at most ~0.09%. The Zn concentrations to which the organisms were exposed (Section 4.2 and Section 4.3) are not affected by this correction, because the toxicity tests were performed with the same concentrated extract analyzed by ICP-MS. In other words, more than 99.9% of the incorporated zinc was not mobilized under the TCLP-based conditions, which are more aggressive than those expected in service (Section 3.1). It should be noted that this estimate should be interpreted as an indicative order of magnitude, not as a value of analytical accuracy. This very low mobilization is consistent with the immobilization of zinc in cementitious matrices as calcium hydroxyzincate and within the C-S-H [9,10,11,12], and with the low leachability of heavy metals from cement-based materials reported by Dell’Orso et al. [12]; it is also in line with the small fractions of incorporated nano-TiO_2_ released from photocatalytic cementitious materials under accelerated or outdoor leaching [13,14]. These results were complemented by FTIR analysis of the freeze-dried leachate extracts and of the pure nano-ZnO powder, used as a spectral reference (Figure 9; Table 5).

In Figure 9 and Table 5, the most relevant observation is that the Zn–O stretching band (400–530 cm^−1^), the most intense band of the nano-ZnO powder (depth of 24.6%, about 2.4 times that of any other band of the material), was not detected in either leachate. The weak feature near 540 cm^−1^ is present in both leachates, including the reference one, and therefore cannot be attributed to ZnO. This result, however, only indicates that crystalline ZnO, if present in the freeze-dried residues, was below the detection capability of the technique in a solid residue dominated by acetate salts and dissolved matrix constituents. Likewise, ICP-MS measures total Zn and cannot distinguish dissolved Zn^2+^ from ZnO nanoparticles or small aggregates able to pass through the filter. Taken together, the two techniques show that the fraction of Zn mobilized was small (at most ~0.07–0.08% of the incorporated Zn) and that any particulate ZnO fraction, if present, was too diluted within the saline residue to produce a detectable spectroscopic signal.

The two strongest bands in the fingerprint region of both leachates, at ~1560–1580 and ~1410–1420 cm^−1^, are assigned to the asymmetric and symmetric COO^−^ stretching modes of acetate [55], which originates from the acetic acid/sodium hydroxide TCLP extraction fluid and was retained in the solid residue after concentration and freeze-drying. The O–C–O bending band at ~640–670 cm^−1^ and the weak C–H stretching bands at ~2850–2920 cm^−1^ are consistent with this assignment. The ~1410–1420 cm^−1^ band may also include a minor contribution from the asymmetric stretching (ν_3_) of carbonate formed by reaction of dissolved Ca^2+^ with atmospheric CO_2_ during concentration of the extract at 60 °C; however, the bending bands characteristic of calcite (~875 and ~712 cm^−1^) were not resolved, indicating that any such contribution is small. The bands at ~1020 and ~810 cm^−1^, also present in both leachates, are consistent with Si–O stretching and Si–O–Si bending of dissolved silicate and amorphous silica species, resulting from partial dissolution of cementitious phases (mainly C-S-H) under the action of the acidic extraction fluid, a phenomenon documented in leaching studies of cementitious matrices [56]; the ~1020 cm^−1^ band may partially overlap with the CH_3_ rocking mode of acetate. Because the spectra of the two leachates share the same set of bands and differ only in intensity, which also depends on the amount of sample analyzed, the FTIR data do not indicate any compositional difference attributable to the incorporation of nano-ZnO.

Finally, although the leached zinc fraction is small, both in absolute terms and relative to the total incorporated mass, the concentration of 0.653 mg/L in the concentrated extract is close to the range in which measurable toxicological effects have been reported for *D. magna* exposed to Zn^2+^ and nano-ZnO [18]. This result highlights the importance of the acute and chronic toxicity assays, since a low leached fraction does not necessarily imply the absence of biological effects on the test organism.

### 4.2. Toxicological Assay with D. magna: Acute

The nonlinear regression parameters (log(agonist) vs. normalized response; variable slope, GraphPad Prism^®^) used to estimate the EC50, 48 h of each test substance are summarized in Table 6.

According to Table 6, the EC50, 48 h values of the two leachates were very similar, with central values of 10.4% *v*/*v* (reference concrete leachate) and 10.5% *v*/*v* (leachate from the concrete with 9% nano-ZnO) and overlapping 95% confidence intervals. Because overlapping confidence intervals do not constitute a formal test [48], the two estimates were compared by the ratio test: the EC50 ratio (nano-modified/reference) was 1.01 (95% CI: 0.87–1.17; *p* = 0.90), indicating no statistically significant difference. The absence of a significant difference does not, by itself, demonstrate equivalence; nevertheless, the 90% CI of the ratio (0.89–1.14) lies entirely within the 0.80–1.25 margin, that is, any difference between the two EC50 values is smaller than about 15%, which is well below the inter-test variability normally accepted for *D. magna* (e.g., the 0.6–2.1 mg/L acceptance range for the reference toxicant K_2_Cr_2_O_7_ in ISO 6341 [45]). Therefore, the incorporation of 9% nano-ZnO (relative to the mass of Portland cement) into the concrete did not detectably increase the acute toxicity of the leachate toward *D. magna*, when compared to the reference concrete without nanoparticles.

The EC50, 48 h of the nano-ZnO powder (4.05 mg/L; 95% CI: 2.01–8.16 mg/L, equivalent to 3.25 mg Zn/L) falls within the toxic band (1–10 mg/L) of the Directive 93/67/EEC scheme [50] and within Acute Category 2 of the UN GHS [52]. Under the EU CLP Regulation, which retains only Acute Category 1 (L(E)C50 ≤ 1 mg/L), this single result would not trigger acute classification, although ZnO as a substance carries a harmonized classification as Aquatic Acute 1 and Aquatic Chronic 1 (Annex VI) [53]. This value is of the same order of magnitude as the range reported in the literature for nano-ZnO toward *D. magna* (~0.76–6.35 mg/L, depending on particle size and degree of agglomeration) [18,19]. For the leachates, the EC50, 48 h values of the concentrated extracts (10.4 and 10.5% *v*/*v*) correspond to original-leachate equivalents of approximately 11.6–13.0% *v*/*v* (reference) and 11.7–13.1% *v*/*v* (nano-modified), considering the CF of 1.11–1.25 (Section 3.1). On this basis, the whole-effluent scheme of Tonkes et al. [51] places both leachates in the slightly toxic band (10–100%), with acute toxic units of TUa ≈ 7.6–8.7 (versus TUa ≈ 9.5–9.6 if the concentrated extracts were taken as the original leachates). Because the CF is ≥1, the values referred to the concentrated extract are conservative, and the classification is the same under both expressions; the comparison between the two leachates is unaffected, because both were produced and concentrated under identical conditions. As stated in Section 3.3, these categories are used only to describe and compare intrinsic toxicity under the tested laboratory conditions.

Cross-referencing with the ICP-MS data shows that, at the dilution corresponding to the EC50, 48 h of the nano-modified leachate (10.5% *v*/*v*), the zinc concentration present would be only 68.6 µg/L (0.653 mg/L × 0.105 = 0.0686 mg/L), a value 11 to 19 times lower than the EC50, 48 h range reported in the literature [18] for Zn^2+^ and nano-ZnO toward *D. magna* (0.76–1.32 mg/L). This margin suggests that dissolved zinc alone is not sufficient to explain the magnitude of the observed effect, and it is more likely that it contributes only partially, together with components of the cementitious matrix. The similarity of the EC50, 48 h values of the two leachates suggests a common origin of their acute toxicity, related to the leached matrix and to the extraction fluid, for example, high ionic strength and alkalinity, dissolved Ca, Na, and K, and acetate from the TCLP fluid, all further enriched by the evaporative concentration step. Because a procedural blank of the extraction fluid was not tested and the major ions were not quantified, these contributions could not be apportioned.

In summary, the results of the acute assay indicate that: (i) the pure nano-ZnO powder is toxic to *D. magna*, within the range expected from the literature for this material; (ii) the fraction of zinc effectively leached from the nano-modified concrete is too low to be, on its own, the explanation for the acute toxicity observed in the leachate; and (iii) this toxicity did not differ significantly from, and fell in the same descriptive category as, the toxicity of the reference leachate. Therefore, under the conditions evaluated, the incorporation of 9% nano-ZnO (relative to the mass of Portland cement) did not produce additional acute toxicity clearly attributable to the nanomaterial, beyond that already inherent to conventional concrete subjected to the same leaching process.

### 4.3. Toxicological Assay with D. magna: Chronic

The results of the chronic toxicity assay (21 days), evaluating longevity, body size, reproduction, and age at first brood, are presented in Table 7, together with the respective lowest observed effect concentrations (LOEC) and no observed effect concentrations (NOEC) for each test substance and endpoint.

Before the results are interpreted, the performance of the controls must be considered. The reproduction criterion (≥60 living offspring per surviving parent at day 21) was not met. The CV of control reproduction was 17.0% in the powder test, but 58.3% in the leachate tests (shared control). OECD TG 211 [46] notes that this CV is generally ≤25%; this value is a performance indicator rather than a validity criterion, but the high CV of the leachate control reduces statistical power. For these reasons, longevity is treated as a valid endpoint, whereas reproduction, body size, and age at first brood are treated as supporting endpoints, interpreted by comparison with the concurrent control under identical conditions, and the reproduction NOEC/LOEC values in Table 7 are indicative.

According to Table 7, the most evident pattern is that none of the test substances affected the age at first brood at the concentrations tested, suggesting that this parameter is relatively insensitive to the three conditions evaluated in this study.

The nano-ZnO powder did not affect body size; however, longevity was significantly reduced at the highest concentration tested (0.4 mg/L), a decrease of −20%. For reproduction, a statistically significant reduction of −36.4% was observed at 0.1 mg/L, but not at the higher concentrations of 0.2 and 0.4 mg/L. Because OECD TG 211 [46] states that concentrations above the LOEC should normally show equal or greater adverse effects, and because the present data do not allow a genuine non-monotonic response to be distinguished from experimental variability, no reproduction LOEC/NOEC was assigned, and the isolated effect at 0.1 mg/L is interpreted with caution. The DLS characterization, performed at 100 mg/L, cannot be extrapolated to these exposure concentrations and was therefore not used to explain this pattern. In fact, at 0.025–0.4 mg/L, well below the solubility of ZnO in freshwater media, nano-ZnO is expected to dissolve largely within the first hours of exposure, so that chronic effects are driven mainly by dissolved zinc [18,20]. In this light, the absence of reproductive impairment at 0.2 and 0.4 mg/L (0.16 and 0.32 mg Zn/L) contrasts with the 21-day reproduction EC50 of 0.112 mg Zn/L reported for nano-ZnO by Adam et al. [20]. Part of this difference may stem from the test medium: M4 contains EDTA, which forms strong complexes with Zn^2+^ and lowers its bioavailability, which is why OECD TG 211 [46] advises against chelator-containing media for metal-containing substances. At the nominal Zn concentrations of the powder test (0.02–0.32 mg Zn/L, i.e., ~0.3–4.9 µM), the low-micromolar EDTA content of M4 may have complexed a substantial fraction of the dissolved zinc, so that the chronic toxicity of the powder is probably underestimated relative to EDTA-free media.

The leachate from the concrete containing 9% nano-ZnO did not cause any adverse effect on longevity, body size, reproduction, or age at first brood up to the highest concentration tested (5 mL/L). The only statistically significant change was a +61.3% increase in reproduction at 5 mL/L. Because this is a stimulatory rather than an adverse response, it was not used to derive a LOEC (Table 7). With the present design, it cannot be established whether this increase reflects a hormetic or compensatory response to a low-intensity stressor [57,58,59] or experimental variability, particularly considering the high variability of the control (coefficient of variation of 58.3%). In any case, this effect is unlikely to be attributable to dissolved zinc: at 5 mL/L (0.5% *v*/*v* of the concentrated extract), the corresponding total zinc concentration would be only 0.653 mg/L × 0.005 = 3.27 µg/L, about one order of magnitude below the chronic effect concentrations reported for nano-ZnO in *D. magna* (e.g., a 21-day reproduction EC10 of 0.030 mg Zn/L [20]). Conversely, the use of M4 may also have lowered the apparent chronic toxicity of the nano-modified leachate: the ~3.27 µg Zn/L (~0.05 µM) contributed at 5 mL/L is far exceeded, on a molar basis, by the EDTA present in M4 [46]. The chronic leachate assay therefore cannot discriminate zinc-specific effects at these levels, and the conclusion that leached zinc is unlikely to cause chronic effects rests on the exposure-based comparison above rather than on the absence of effects in the assay. In original-leachate terms, the highest chronic concentration (5 mL/L of the concentrated extract) corresponds to ~5.6–6.3 mL/L of the original leachate.

In the reference concrete leachate (data from [16]), the only statistically significant effect observed was a slight, though statistically significant, reduction (−5.1%) in body size at a concentration of 4 mL/L.

In the chronic assays, effects were detected at concentrations well below the respective EC50, 48 h values: the reduction in body size caused by the reference leachate occurred at 4 mL/L, about 26 times below its EC50, 48 h (104/4 ≈ 26), and the reduction in longevity caused by the nano-ZnO powder occurred at 0.4 mg/L, about 10 times below its EC50, 48 h (4.05/0.4 ≈ 10). The isolated decrease in reproduction observed for the powder at 0.1 mg/L (about 40 times below the EC50, 48 h; 4.05/0.1 ≈ 40) was not assigned as a LOEC and, as discussed above, cannot be distinguished from experimental variability; it is therefore not considered further here. These results illustrate that acute assays alone may not capture effects that emerge under longer exposure.

In summary, the incorporation of 9% nano-ZnO into the concrete (relative to the mass of Portland cement) did not produce any adverse chronic effect detectable with the present design in the leachate up to 5 mL/L, and the nano-modified leachate did not show a chronic effect profile more severe or broader than that of the leachate from conventional concrete. This result is consistent with the acute results and with the low Zn mobilization measured by ICP-MS. However, because the physical form of the leached zinc was not determined and the chronic assays have limited sensitivity, did not meet the OECD TG 211 reproduction validity criterion, and were performed in EDTA-containing medium, these results support the absence of additional toxicity under the tested conditions, rather than demonstrating that the bioavailability of nano-ZnO is suppressed by the cementitious matrix.

### 4.4. Limitations of the Study

The main limitations of this study are as follows: (i) the TCLP-based extraction of crushed concrete is an accelerated and conservative laboratory condition, which exposes the internal cement paste to an aggressive and buffered fluid; it does not represent the surface wash-off of in-service pavements or the release of abrasion-derived particles; (ii) the physical form of the leached zinc (dissolved, complexed, or particulate) was not determined, because ICP-MS measures total Zn and FTIR has a limited detection capability for a minor crystalline phase in a salt-dominated residue; and (iii) the chronic assay was based on, but not fully compliant with, OECD TG 211; consequently, the chronic results, particularly for reproduction, are supporting rather than conclusive evidence.

## 5. Conclusions

This study evaluated whether the incorporation of nano-ZnO, at a proportion of 9% relative to the mass of Portland cement, increases the toxicity toward *D. magna* of leachates obtained from pavement concrete under accelerated laboratory leaching, compared to conventional concrete.

Under the conditions evaluated, the results did not indicate additional toxicity. At most ~0.07–0.08% of the incorporated zinc was mobilized under TCLP-based conditions (653 ± 76 µg/L of total Zn in the concentrated extract), and no Zn–O band was detected by FTIR in the freeze-dried leachates; however, neither technique establishes the physical form of the leached zinc. In the acute assay, the EC50, 48 h of the nano-modified leachate (10.5% *v*/*v* of the concentrated extract) did not differ significantly from that of the reference leachate (10.4% *v*/*v*; EC50 ratio 1.01, 95% CI 0.87–1.17) and was not attributable to dissolved zinc alone, whose concentration at the EC50, 48 h point was 11 to 19 times below the toxic range described in the literature for Zn^2+^/nano-ZnO; the acute toxicity of both leachates is therefore most likely associated with components of the leached cementitious matrix and of the extraction fluid, although their individual contributions could not be apportioned, as neither a procedural blank of the extraction fluid nor the major ions were analyzed. In 21-day assays based on OECD TG 211, the nano-modified leachate caused no adverse effect up to the highest concentration tested, whereas the reference leachate caused a slight reduction in body size; however, because these assays did not meet the reproduction validity criterion of the guideline and were performed in EDTA-containing M4 medium, the chronic results are regarded as supporting rather than conclusive evidence. Thus, the incorporation of 9% nano-ZnO did not produce additional acute toxicity, and no additional chronic toxicity was detected, under the tested conditions.

It is important to emphasize that these conclusions are limited to the test organism, the specific nature of the nano-ZnO, the nano-ZnO content added to the cement, the concrete mix design, and the experimental conditions employed here, in particular the TCLP-based leaching of crushed concrete, which is an accelerated, conservative laboratory condition and not a representation of rainfall runoff from in-service pavements. They should not be directly generalized to other aquatic organisms, concrete formulations, or environmental exposure scenarios, nor interpreted as an environmental risk assessment. Future studies should prioritize: (i) surface-controlled leaching of monolithic specimens (tank or dynamic surface leaching tests) and field runoff monitoring, including abrasion-derived particles; (ii) fractionation of the leached zinc (e.g., ultrafiltration or single-particle ICP-MS) and quantification of major ions, with procedural blanks of the extraction fluid; (iii) chronic tests fully compliant with OECD TG 211; and (iv) other aquatic species and trophic levels.

## Figures and Tables

**Table 1:** Characteristics of the ZnO nanoparticles according to the manufacturer [24].

Characteristic	Result
Crystal Structure	Wurtzite
Average Size	~20 nm
Morphology	Spherical to Sub-spherical
Specific Surface Area	≥40 m^2^/g
Density	5.6
pH	6.5–7.5
Color	White
Loss on Drying (110 °C/2 h)	≤1% by Weight
Loss on Ignition (850 °C/2 h)	≤3% by Weight

**Table 2:** Chemical composition, expressed as oxide content, of the ZnO nanoparticles.

Oxides	%
ZnO	98.8 ± 0.14
P_2_O_5_	0.86
TiO_2_	0.13
CuO	0.09
Nd_2_O_3_	0.08
Cr_2_O_3_	0.04

**Table 3:** Zeta potential, effective diameter, polydispersity index, and pH of the ZnO nanoparticles.

Parameter	Ultrapure Water	ISO	M4
Zeta Potential (mV)	11.20 ± 0.12	−2.20 ± 0.40	−1.25 ± 0.07
Effective Diameter (nm)	517.65 ± 11.07	1690.11 ± 14.88	1121.55 ± 0.77
Polydispersity Index	0.12 ± 0.12	0.27 ± 0.03	0.26 ± 0.02
pH	7.39	7.52	7.60

**Table 4:** Concentrations used in the chronic toxicity assays with *D. magna*.

Nano-ZnO (mg/L)	Reference Concrete Leachate (mL/L)	Leachate from Concrete with 9% Nano-ZnO (mL/L)
0	0	0
0.025	0.25	0.1
0.05	0.5	0.5
0.1	1.0	1.0
0.2	2.0	5.0
0.4	4.0	-

**Table 5:** Main infrared absorption bands of the nano-ZnO powder and the freeze-dried leachates (values are band depths (% transmittance) relative to the local baseline. n.d., not detected).

Wavenumber (cm^−1^)	Powder Nano-ZnO	Leachate		Assignment
		Concrete with 9% Nano-ZnO	Reference Concrete	
~3410–3440	4.8%	2.6%	10.0%	O–H stretching (adsorbed water, hydroxyl groups, silanols).
~1560–1580	n.d.	3.4%	6.8%	Asymmetric COO^−^ stretching, νas(COO^−^), of acetate from the TCLP extraction fluid.
~1385–1420	10.1%	2.8%	6.3%	Powder (1385 cm^−1^): asymmetric C–O stretching, ν_3_(CO_3_^2−^), of surface carbonate. Leachates (~1415 cm^−1^): symmetric COO^−^ stretching, νs(COO^−^), of acetate, with a possible minor ν_3_(CO_3_^2−^) contribution.
~1020	n.d.	1.0%	4.1%	Si–O stretching of dissolved silicate species, overlapping with CH_3_ rocking of acetate.
~810	n.d.	1.6%	4.9%	Si–O–Si bending (amorphous silica/silicate species).
~640–670	n.d.	1.9%	5.7–6.0%	O–C–O bending, δ(OCO), of acetate.
~400–530	24.6%	n.d.	n.d.	Zn–O stretching (wurtzite lattice).

**Table 6:** Nonlinear fitting parameters for determination of the EC50, 48 h in *D. magna*. Data for the reference-concrete leachate were previously published in [16] and are reproduced here for comparison.

Parameter	Powder Nano-ZnO	Leachate	
		Concrete with 9% Nano-ZnO	Reference Concrete
EC50, 48 h	4.05 mg/L	105 mL/L (10.5% *v*/*v*)	104 mL/L (10.4% *v*/*v*)
95% CI	2.01–8.16 mg/L	93.3–118.2 mL/L	95.3–113.6 mL/L
Hill Slope	0.85	3.53	3.24
R^2^	0.730	0.974	0.986
pH	7.92	7.62	7.65

**Table 7:** Effects of nano-ZnO powder, leachate from concrete containing 9% nano-ZnO, and leachate from reference concrete on the longevity, size, reproduction, and age at first brood of the test organism exposed for 21 days. Values are expressed as mean ± standard deviation. Data for the reference-concrete leachate were previously published in [16] and are reproduced here for comparison.

Nano-ZnO Powder				
Concentration (mg/L)	Longevity (%)	Size (mm)	Reproduction (Neonates/Brood)	Age at First Brood (Days)
0	100	4.017 ± 0.44	4.766 ± 0.81	11.60 ± 2.87
0.025	100	3.858 ± 0.12	4.850 ± 0.74	11.70 ± 2.66
0.05	100	3.881 ± 0.09	4.600 ± 1.19	10.80 ± 1.32
0.1	100	3.871 ± 0.17	3.030 ± 0.89 *	12.60 ± 3.44
0.2	100	3.806 ± 0.12	4.370 ± 1.03	12.10 ± 2.13
0.4	80 *	4.176 ± 0.24	4.213 ± 1.19	13.00 ± 2.33
LOEC mg/L	0.4	>0.4	n.a. ^a^	>0.4
NOEC mg/L	0.2	>0.4	n.a. ^a^	>0.4
**Leachate from Concrete Containing 9% Nano-ZnO**				
**Concentration (mL/L)**	**Longevity (%)**	**Size (mm)**	**Reproduction (neonates/brood)**	**Age at First Brood (days)**
0	90	2.860 ± 0.13	3.533 ± 2.06	12.88 ± 1.13
0.1	90	2.752 ± 0.27	4.367 ± 1.22	14.33 ± 2.23
0.5	100	2.815 ± 0.13	4.410 ± 1.88	13.56 ± 1.81
1	80	2.877 ± 0.04	3.900 ± 2.34	12.50 ± 1.60
5	100	2.862 ± 0.09	5.700 ± 1.13 †	12.00 ± 1.15
LOEC (mL/L)	>5	>5	>5 ^b^	>5
NOEC (mL/L)	>5	>5	>5 ^b^	>5
**Leachate from Reference Concrete**				
**Concentration (mL/L)**	**Longevity (%)**	**Size (mm)**	**Reproduction (neonates/brood)**	**Age at First Brood (days)**
0	90	2.860 ± 0.13	3.533 ± 2.06	12.88 ± 1.13
0.25	100	2.766 ± 0.11	4.090 ± 1.97	12.70 ± 0.83
0.5	80	2.777 ± 0.13	4.550 ± 1.17	11.38 ± 1.30
1	90	2.740 ± 0.13	4.511 ± 1.09	13.00 ± 1.22
2	100	2.780 ± 0.09	4.510 ± 0.54	12.70 ± 2.40
4	100	2.714 ± 0.11 *	3.560 ± 1.55	12.89 ± 1.16
LOEC (mL/L)	>4	4	>4	>4
NOEC (mL/L)	>4	2	>4	>4

* Statistically significant decrease relative to the control (*p* < 0.05). † Statistically significant increase relative to the control (*p* < 0.05), not considered an adverse effect. ^a^ Not assigned: the significant decrease at 0.1 mg/L was not accompanied by equal or greater effects at 0.2 and 0.4 mg/L (see text). ^b^ No adverse effect up to the highest concentration tested; the only significant change was a stimulatory response (†). n.a., not assigned.

## Data Availability

The original contributions presented in this study are included in the article. Further inquiries can be directed to the corresponding author.
